# Adaptive optics retinal imaging in patients with usher syndrome

**DOI:** 10.3389/fopht.2024.1349234

**Published:** 2024-05-28

**Authors:** Melanie Kempf, Susanne Kohl, Krunoslav Stingl, Fadi Nasser, Katarina Stingl, Friederike C. Kortuem

**Affiliations:** ^1^ University Eye Hospital, Center for Ophthalmology, University of Tuebingen, Tuebingen, Germany; ^2^ Center for Rare Eye Diseases, University of Tuebingen, Tuebingen, Germany; ^3^ Institute for Ophthalmic Research, Center for Ophthalmology, University of Tuebingen, Tuebingen, Germany; ^4^ University Eye Hospital, University of Leipzig, Leipzig, Germany

**Keywords:** adaptive optics imaging, usher syndrome, degenerative retinal disease, multimodal degenerative retinal disease, multimodal imaging, cone mosaic, electroretinogram

## Abstract

**Purpose:**

To determine the structure of the cone photoreceptor mosaic in the macula in eyes with retinitis pigmentosa related to Usher syndrome using adaptive optics fundus (AO) imaging and to correlate these findings with those of the standard clinical diagnostics.

**Methods:**

Ten patients with a genetically confirmed retinitis pigmentosa in Usher syndrome due to biallelic variants in MYO7A or USH2A were enrolled in the study. All patients underwent a complete ophthalmological examination including best corrected visual acuity (BCVA), spectral-domain optical coherence tomography (SD-OCT) with fundus autofluorescence photography (FAF), full-field (ffERG) and multifocal electroretinography (mfERG) and Adaptive Optics Flood Illuminated Ophthalmoscopy (AO, rtx1™, Imagine Eyes, Orsay, France). The cone density was assessed centrally and at each 0.5 degree horizontally and vertically from 1–4 degree of eccentricity.

**Results:**

In the AO images, photoreceptor cell death was visualized as a disruption of the cone mosaic and low cone density. In the early stage of the disease, cones were still visible in the fovea, whereas outside the fovea a loss of cones was recognizable by blurry, dark patches. The blurry patches corresponded to the parafoveal hypofluorescent ring in the FAF images and the beginning loss of the IS/OS line and external limiting membrane in the SD-OCT images. FfERGs were non-recordable in 7 patients and reduced in 3. The mfERG was reduced in all patients and correlated significantly (p <0.001) with the cone density. The kinetic visual field area, measured with III4e and I4e, did not correlate with the cone density.

**Conclusion:**

The structure of the photoreceptors in Usher syndrome patients were detectable by AO fundus imaging. The approach of using high-resolution technique to assess the photoreceptor structure complements the established clinical examinations and allows a more sensitive monitoring of early stages of retinitis pigmentosa in Usher syndrome.

## Introduction

Usher syndrome (USH) is an autosomal recessive disease characterized by hearing loss and retinitis pigmentosa (RP) in some subtypes. In addition, patients can present with vestibular and olfactory deficits (1). The syndrome is the most common genetic cause of deaf-blindness (1, 2) and was first recognized and described by Albrecht von Graefe in 1858 (3) and further investigated by Charles Usher in 1914. Functionally, RP is characterized by impaired night vision, progressive loss of the visual field and a reduced visual acuity due to the progressive degeneration of the photoreceptors. Fundus features include blood vessel attenuation, disc pallor, cystoid macular edema and peripheral bone spicule derived from the retinal pigment epithelium (RPE) changes (4). Usher syndrome is subdivided into four clinical subtypes (USH1, USH2, USH3 and USH4) depending on the severity and time of onset of auditory, vestibular and visual symptoms (5). At least 9 causative genes have been found and identified to be responsible for Usher syndrome: Five for USH1 (*MYO7A, USH1C, CDH23, PCDH15*, and *USH1G*), three for USH2 *(USH2A*, *ADGRV1*, and *WHRN*) and one for USH3 (*CLRN1*). However, other genes and loci have been proposed and discussed to be related to Usher syndrome (6).

Hearing aids or cochlear implants can compensate the hearing loss, but so far there is no approved causal genetic treatment for the RP in Usher syndrome. However, over the last several years research on Usher syndrome has increased, with studies on gene and cell therapies providing hope for an efficient prevention of the sensory loss (7). Nevertheless, more studies on the natural history of the disease are required to aid planning clinical trials. Reliable and sensitive tools need to be tested and developed to evaluate the effect of experimental treatments.

With the availability of adaptive optics (AO) fundus imaging, the non-invasive visualization of the photoreceptor mosaic on a cellular level became possible (8, 9), and abnormalities in individual cone photoreceptor mosaic in patients with various retinal diseases could be studied (10–18). AO technologies have also been combined with optical coherence tomography (AO-OCT), fundus photography and scanning-laser ophthalmoscopy (AO-SLO), that increased the sensitivity and resolution and thus provided new insights into the effects of retinal degenerations (12–14, 18–24).

In this study we used flood illuminated adaptive optics (FI-AO) camera (rtx1™), an advanced high-resolution fundus imaging method to quantify the photoreceptor mosaic in RP associated with Usher syndrome. Additionally, we compared and correlated the results with those of spectral-domain optical coherence tomography (SD-OCT), fundus autofluorescence photography (FAF), semiautomatic kinetic visual field testing, full-field (ffERG) and multifocal electroretinography (mfERG) that are used for clinical ophthalmological diagnostics in our clinical routine for inherited retinal dystrophies. The aim was to explore the use of high-resolution FI-AO for diagnosis and monitoring of disease progression.

## Materials and methods

Ten patients with a clinical diagnosis of RP with Usher syndrome and likely biallelic variants in *MYO7A* (Usher syndrome type 1) or *USH2A* (Usher syndrome type 2) who presented for follow-ups at the University Eye Hospital, Tübingen were included in the study (**Table 1**). Exclusion criteria included the inability of a moderate fixation, macular edema, central media opacity such as but not only cataract. In addition, patients with a history of any systemic disease (e.g. hypertension) or other eye complications were excluded. The examinations were approved by the institutional Ethics Committee and adhered to the Declaration of Helsinki. All subjects were informed about the consequences of the procedure and informed consent was received from all subjects. Statistical analysis was performed using SPSS (Version 27.0 SPSS Inc. Chicago, IL) and the graphs were created using JMP16 (SAS institute Inc., Cary, NC, USA).

**Table 1:** Clinical data of all patients including genetic findings, age, visual acuity, kinetic perimetry, mf ERG, and description of fundus, SD-OCT and FAF.

Patient	Gene mutations	Age	Eye	BCVA(feet)	Kinetic visual field area (deg^2^)		FI-AO foveal cones (cones/deg^2^)	Full-field ERG		Multifocal ERG	Fundus photography	SD-OCT	FAF
					III4e	I4e		photopic	scotopic				
**1**	*MYO7A: c.3719G>A; p.R1240Q homozygous*	16	OD	20/25	395,0	230,4	609	Severely reduced		Rings 1,2,3 reduced, rings 4, 5 ND	Optic disc drusen, macula with normal reflex, narrow vessels, few pigmentations in periphery	Small cysts in the internal layers, normal photoreceptor layer in macula	Normal fovea with hyperfluorescent ring, hyperfluorescent optic disc drusen
			OS	20/25	369,9	183,7	1171						
**2**	*MYO7A: c.494C>T;p.T165M heterozygous + c.3610C>A;p.P1204T heterozygous*	60	OD	20/25	3023,8	2369,6	ND	Reduced	Mildly reduced	Mildly reduced	Pallor of optic disc, macula with normal reflex, narrow vessels, pigmentations in periphery	Normal layers in macula in OD	Normal centrally
			OS	20/33	3190,2	2601,6							
**3**	*USH2A: c.13010C>T;p.T4337M heterozygous + Deletion Exon 23 heterozygous*	43	OD	20/200	126,2	NA	ND	ND (2013)		ND (2013)	Waxy optic disc, macula without reflex, narrow vessels, pigmentations in periphery	Cysts in the internal layers of the retina (large cysts in OS), atrophy in the photoreceptors and RPE in macula	Hyperfluorescence in fovea surrounded by hypofluorescent area up to arcades
			OS	20/200	146,5								
**4**	USH2A: c.9270C>A;p.C3090* heterozygous + c.11864G>A;p.W3955* heterozygous	26	OD	20/33	5921,5	110,5	1179	ND (2013)		Rings 1,2,3 severely reduced, rings 4,5 ND	Pallor of optic disc, macula with normal reflex, narrow vessels, pigmentations in periphery	Normal layers in macula	Bull’s eye maculopathy
			OS	20/25	4516,0	150,2	968						
**5**	MYO7A: c.2878G>T;p.E960* heterozygous + c.4852+1G>A; p.? heterozygous	25	OD	20/20	307,1	125,6	1362	ND (2014)		Rings 1,2,3 mildly reduced, rings 4,5 ND	Optic disc drusen, macula with rest reflex, narrow vessels, pigmentations in periphery	Cysts in the internal layers, epiretinal membrane and normal photoreceptor layer in macula	Bull’s eye maculopathy, hyperfluorescent optic disc drusen
			OS	20/25	352,4	133,6	1294						
**6**	USH2A: c.653T>A;p.V218E heterozygous + c.11105G>A;p.W3702* heterozygous	16	OD	20/33	6423,1	321,2	728	Severely reduced	ND	Rings 1,2,3 mildly reduced, rings 4,5 ND	Normal optic disc, macula with normal reflex, normal vessels, few pigmentations in periphery	Normal layers in macula, with small cysts in the internal layers of the retina in OS	Normal fovea with hyperfluorescent ring
			OS	20/33	5151,1	297,5	1125						
**7**	*USH2A: c.7595-2144A>G; p.? homozygous *	54	OD	20/400	ND	ND	414	NA		ND (2014)	Waxy optic disc, macula without reflex, narrow vessels, pigmentations in periphery	Severe atrophy in the photoreceptors and RPE layers, mild epiretinal membrane	Hyperfluorescent fovea surrounded by hypofluorescent area and then incomplete hyperfluorescent ring, then multi hypo patchy areas up to arcades
			OS	20/160			611						
**8**	*USH2A: c.1841-2A>G;p.? heterozygous + c.12448A>G; p.T4150A heterozygous*	58	OD	20/40	271,0	NA	214	ND (2017)		ND (2018)	Waxy optic disc, macula without reflex, narrow vessels, pigmentations in periphery	Few small cysts in the internal layers, remains of photoreceptor layer	Bull’s eye maculopathy, arcades with hypo patchy areas
			OS	20/63	177,1		NA						
**9**	USH2A: c.4776+2T>C; p.? heterozygous + c.14131C>T; p.Q4711*	46	OD	20/20	64,3	3,9	894	ND (2011)		ND (2016)	Pallor of optic disc, macula with reflex, narrow vessels, pigmentations in periphery	Normal layers in the fovea, epiretinal membrane	Bull’s eye maculopathy, arcades with hypo patchy areas
			OS	20/25	50,5	4,3	462						
**10**	USH2A: c.1036A>C;p.N346H heterozygous + c.11864G>A;p.W3955* heterozygous	45	OD	LP	ND	ND	NA	ND (2011)		ND (2011)	Waxy optic disc, macula without reflex, narrow vessels, no pigmentations, multi atrophic chorioretinal regions in the far periphery	Severe atrophy in photoreceptors and RPE layers in OD with remains of photoreceptor layer in the fovea; few small cysts in the internal layers of the retina in OS, epiretinal membrane in both eyes	Bull’s eye maculopathy, arcades with hypo patchy areas (limited image quality)
			OS	20/40			429						

LP, light perception; ND, not detectable; NA, not available.

### Ophthalmologic examinations

All subjects underwent a comprehensive ophthalmological examination, including best-corrected visual acuity (BCVA), indirect ophthalmoscopy, slit-lamp examination, semiautomated kinetic visual field testing with the Octopus 900 (Haag-Streit, Wedel, Germany) using the III4e (4 mm^2^, 320 cd/m^2^, white stimulus) on a white standardized background (10 cd/m^2^) and I4e target (0.25 mm^2^, 320 cd/m^2^, white stimulus) mark. Full-field (ffERG) and multifocal ERG (mfERG) were recorded using the Espion E2 and E3 (Diagnosys LCC, Cambridge, UK) according to the standards of the International Society for Clinical Electrophysiology of Vision (ISCEV) (25, 26).

Retinal imaging was performed with color fundus photography (Zeiss FF450 IR Fundus Camera, Carl Zeiss Meditec, Jena, Germany), Adaptive Optics Flood Illumination Ophthalmoscopy (rtx1™, Imagine Eyes, Orsay, France) spectral domain optical coherence tomography (SD-OCT) with fundus autofluorescence photography (FAF, Spectralis^®^ HRA+OCT system Heidelberg Engineering GmbH, Heidelberg, Germany). A volume scan, a horizontal and vertical line scan through the central fovea was acquired with the SD-OCT.

In AO, a montage covering the central area of approximately 8 x 8 degree was combined from single images. The montage was either automatically created by the inbuilt software (i2k Retina Pro Version 3.1.0) provided by the manufacturer or manually assigned by a single observer. The cone density was analyzed at each 0.5 degree of the horizontal and vertical line from 1–4 degree of eccentricity, as well as at the central foveola. Cones were identified in a region of interest (ROI), an 80x80 pixels sampling area, corresponding to an area of approximately 63 x 63 µm on the retina and were analyzed using a custom semiautomated software provided by the manufacturer (AOdetect, version 3.0, Imagine Eyes, Orsay, France) that located the cones automatically. Non-waveguiding cones could not be counted by the program. The count was then manually assigned by the observer. Especially in the strongly degenerated areas a manually assessment and correction was necessary. The axial length was not measured, therefore the cone density is expressed in number of cones per degree^2^ (visual units), where the eye length is not taken into account in the calculation. Due to poor image quality the AO-FIO images from 4 of the 18 eyes had to be excluded from analysis.

### Genetic testing

All patients had a genetically confirmed Usher syndrome with likely biallelic mutations in the *MYO7A* or *USH2A* genes (see **Table 1**). Three patients suffered from Usher syndrome type I (USH1), seven patients from Usher syndrome type II (USH2).

## Results

The clinical characteristics and genetic profiles of the patients are summarized in **Table 1**, along with a short description of the fundus photography, imaging and ERG results. The patients were between 16 and 60 years old (**Table 1**). All USH1 patients had cochlea implants and USH2 patients had hearing aids. The best-corrected visual acuity (BCVA) ranged from 20/400 (feet) to 20/20 (feet). One eye had light perception. Here, only limited examinations were possible. The mean BCVA was 0.25 ± 0.43(logMAR) for the right eye and 0.24 ± 0.52 (logMAR) for the left, which is approximately equivalent to 20/40.

### Fundus photography

**Figure 1:**
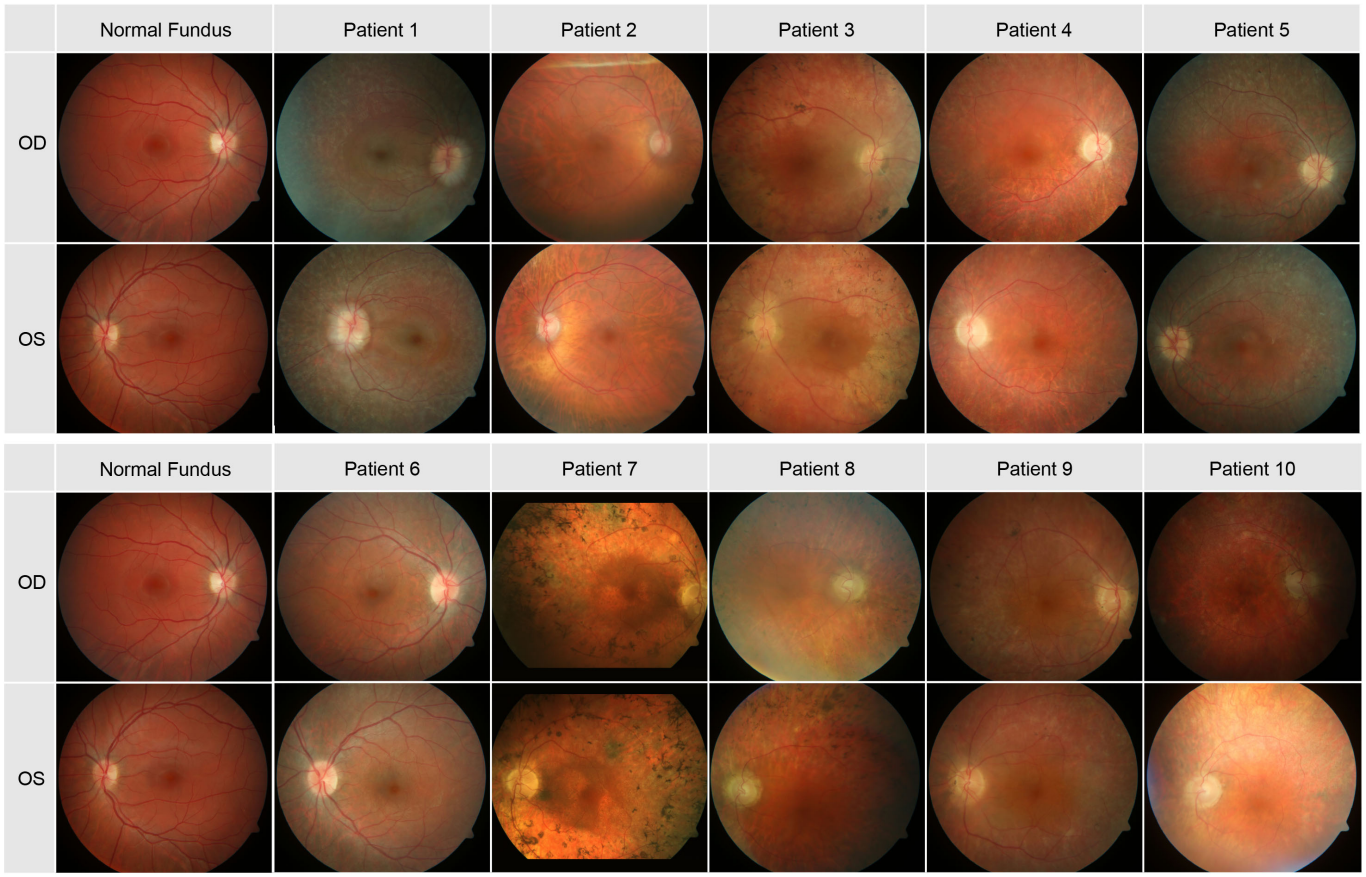
Color photos of the central pole of all 10 patients.

In **Figure 1** the fundus photos of the 10 patients are shown. Nine patients had RPE changes with pigmentations (i.e. bone spicules) in the periphery, and one patient (Patient 10) had no pigmentations in the fundus but displayed multiple atrophic chorioretinal lesions in the far periphery. The macula was without reflex in patients 3, 5, 6, 7, 8 and 10 and with good reflex in patients 1, 2, 4 and 9. Most patients had narrow vessels, except patient 6 who had a normal vasculature. The optic disc appearance was normal in patient 6, pale in patients 2, 4 and 9 and waxy in patients 3, 7, 8 and 10. Two patients (Patients 1 and 5) had optic disc drusen.

### OCT imaging

**Figure 2:**
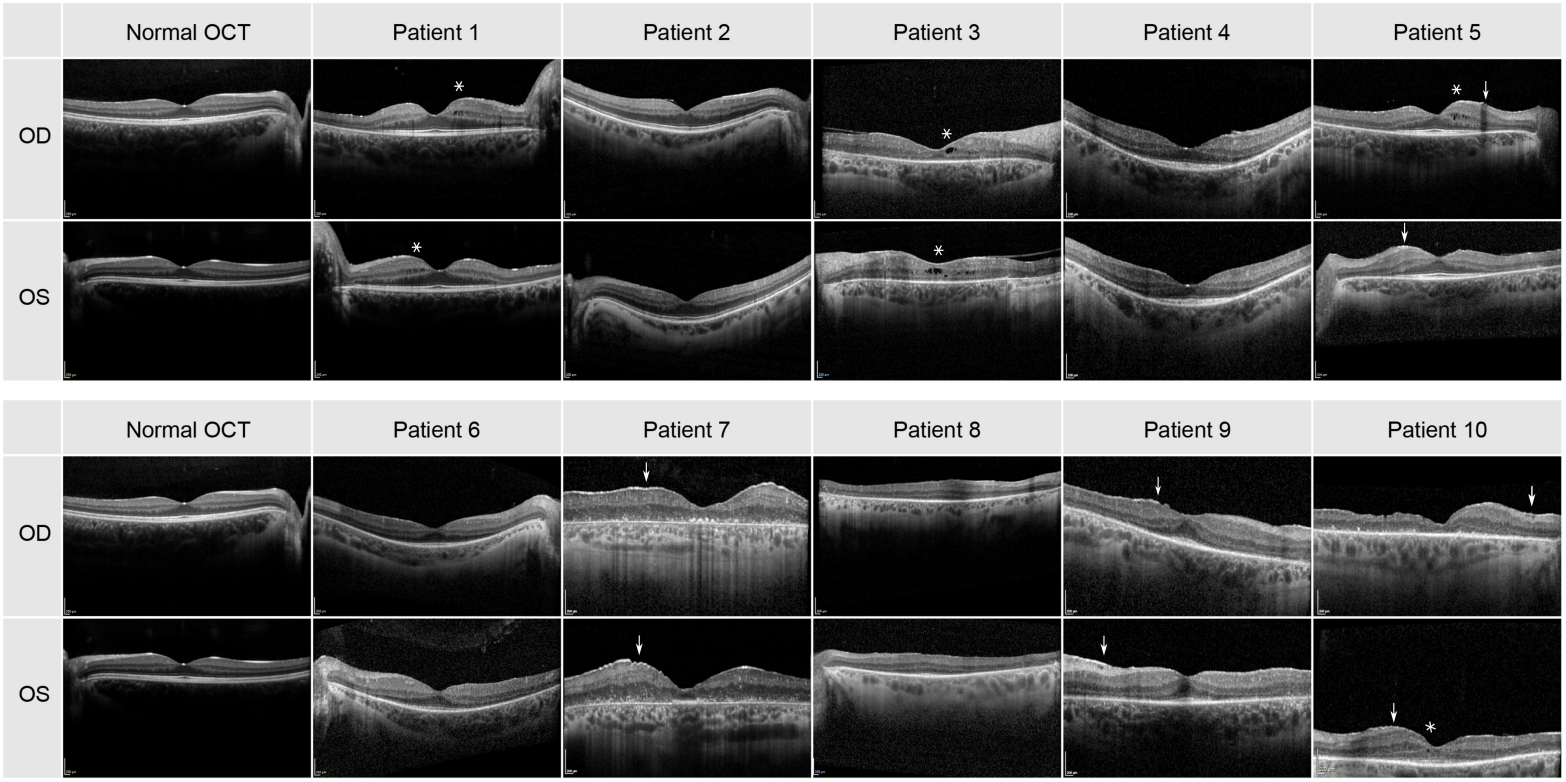
Transfoveal horizontal line scan of the central macula for all 10 patients. Cysts are highlighted by an asterisk and the epiretinal membrane by an arrow.

The transfoveal horizontal line scan of all patients can be seen in **Figure 2**. Small cysts in the internal layers in both eyes of patients 1, 5 and 8 were found, as well as in the left eye of patients 6 and 10. The cysts were large in both eyes in patient 3 (see asterisks in **Figure 2**). A discrete epiretinal membrane was apparent in both eyes of patients 5, 7, 9 and 10 (see arrows in **Figure 2**). These membranes did not have an effect on the foveal contour and depression. A normal photoreceptor layer in the macula of both eyes was seen in patients 1, 2, 4, 5, 6 and 9, and atrophy in the photoreceptors and RPE in both eyes of patients 3 and 7. In patient 8 the SD-OCT images had a residual photoreceptor layer in the fovea but, the foveal contour was altered due to residual macular edema. The SD-OCT of patient 10 showed severe atrophy in photoreceptor and RPE layers in the right eye, but with a residual photoreceptor layer in the area of the fovea in the left eye.

### Fundus autofluorescence

**Figure 3:**
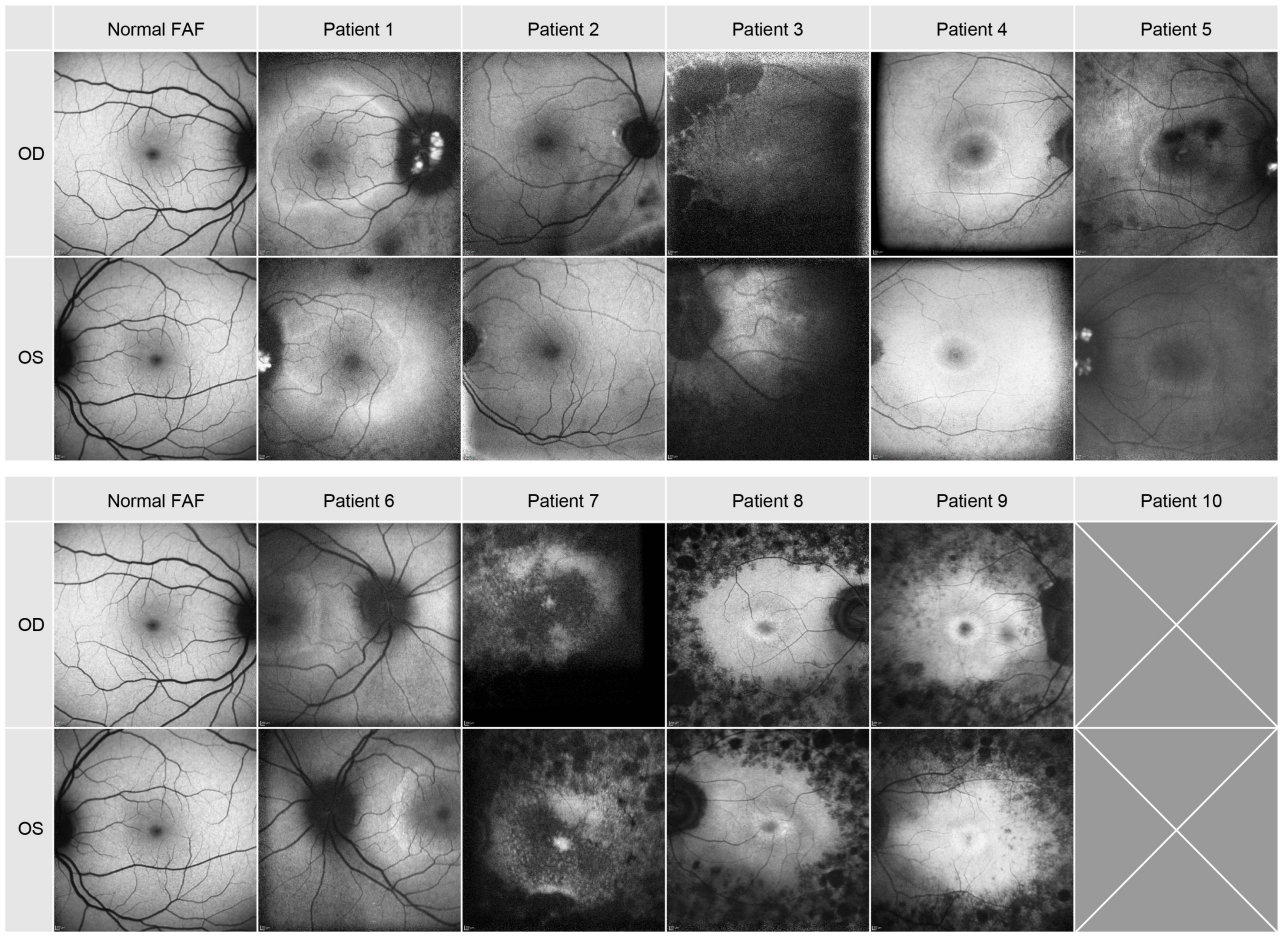
Autofluorescence images of the posterior pole of all patients.

The FAF images are depicted in **Figure 3** and described in **Table 1**. A hypofluorescent center in both eyes of patients 4, 5 8, 9 and 10 with a hyperfluorescent ring (Bull’s eye maculopathy) could be seen and almost normal fovea with a hyperfluorescent ring around it in both eyes of patients 1 and 6. Patient 3 showed FAF hyperfluorescence in the fovea (more in the left eye), which was surrounded by a hypofluorescent area up to the arcades in both eyes. In patients 1 and 5, a hyperfluorescent optic disc was found in both eyes. In patient 7, the FAF showed both hyperfluorescent and hypofluorescent areas from the fovea up to arcades. Patient 1 and 5 had both hyperfluorescent drusen at the optic disc. Only patient 2 showed a normal FAF appearance in the fovea of both eyes.

### Electroretinograms

The scotopic and photopic full-field ERG were not recordable in both eyes in the majority of the patients (patients 3, 4, 5, 7, 8, 9, 10). Patient 1 showed severely reduced amplitudes in the scotopic and photopic ERG and patient 2 had reduced amplitudes in the photopic ERG and mildly reduced amplitudes in the scotopic ERG. Furthermore, the scotopic ERG was undetectable in both eyes of patient 6 and the photopic ERG showed only small remaining amplitudes.

The multifocal ERG was also not detectable. In 50% of the patients (patient 3, 7, 8, 9, 10). Patients 1, 4, 5, 6 showed reduced amplitudes in the first three inner rings and no remaining amplitudes in the two outer rings. Compared to normal values patient 2 showed only mildly reduced responses.

### Visual field

The visual field areas delimited by the Goldmann III4e target were severely reduced in all 10 patients. Patient 7 and 10 could not identify the III4e stimulus (**Table 1**). In two other patients (patient 3, 8) the visual field was not measured with the I4e target. The kinetic visual field area in degree^2^ tested by the III4e ranged from 6423 to 0 and from 2602 to 0 tested by the I4e.

### Adaptive optics

**Figure 4:**
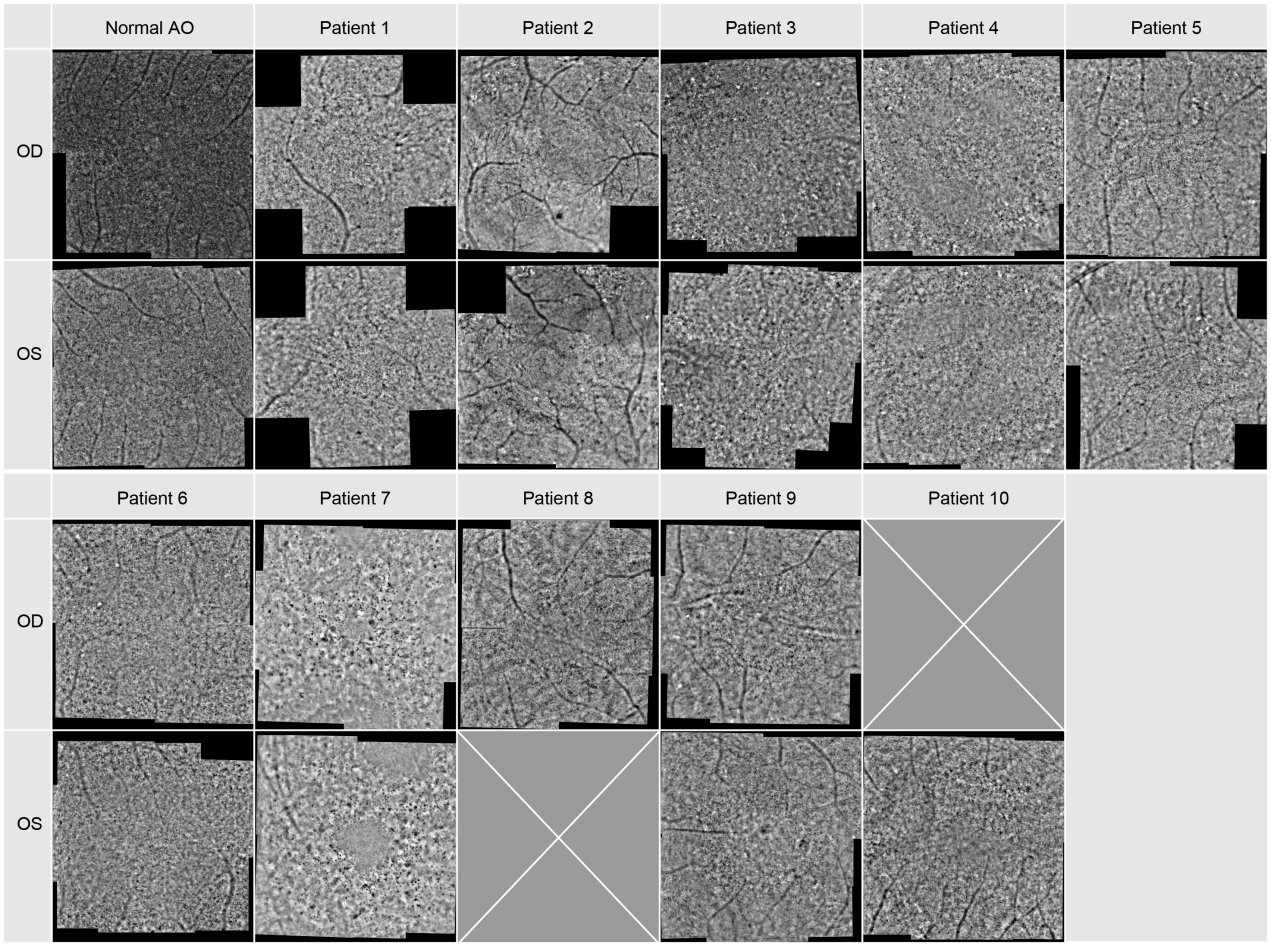
Adaptive optics images of the central macular region. Image montage covers an area of approx. 8x8 degrees for each patient, for Patient 1 it covers only 8 degrees in the horizontal and vertical meridian.

In all patients FI-AO images were obtained. These are depicted in **Figure 4**. In patient 1 only five images per eye were taken and in patients 8 and 10 images of only one eye were acquired. Almost all eyes demonstrated a cone mosaic pattern with some dark patchy areas (hypo-reflective blurred cone-like structures). In patient 1 and 6 the cone mosaic appeared most preserved. In both eyes of patient 7 there was no recognizable mosaic: The image is marked by dark patchy and bright white areas, i.e. hyper- and hyporeflective areas. Only some single waveguiding cones were observable in the whole montage.

When comparing the pattern of these dark blurry regions with the images of the SD-OCT and FAF imaging displayed in **Figures 2**, **3**, they were corresponding to the areas in which cone degeneration is evident i.e. by the loss of IS/OS border and external limiting membrane in **Figure 2** and by the hypofluorescent ring in **Figure 3**.

In patients 2 and 3, only images with a poorer quality could be obtained.

**Figure 5:**
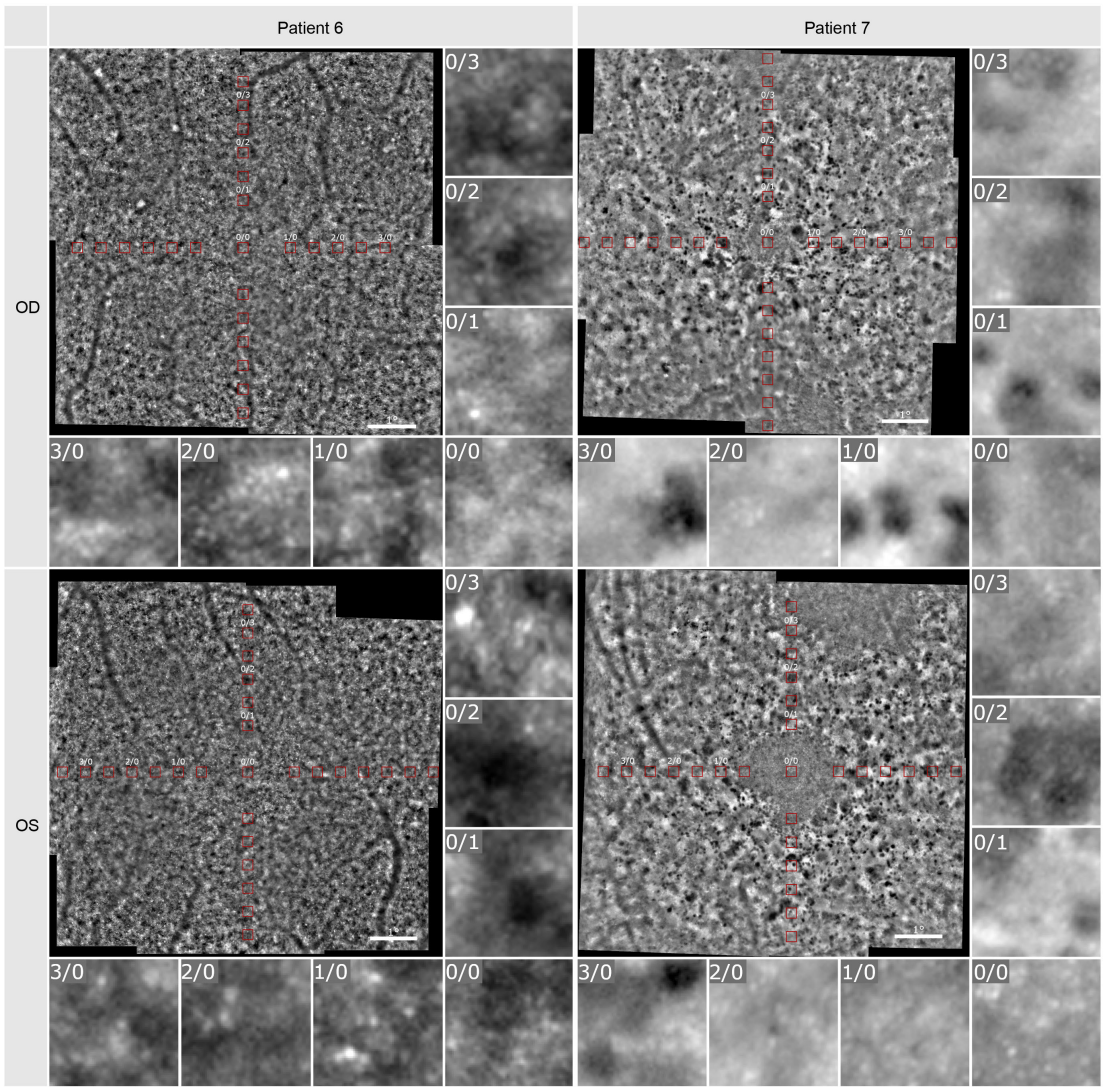
Adaptive optics imaging of patient 6 and 7. The red boxes visualize the positions at which cell density was measured. Seven regions of interest in 80x80 pixels (foveal [0/0] and 1-, 2-, 3-degree nasal and superior) are shown in a larger magnification.

**Figure 6:**
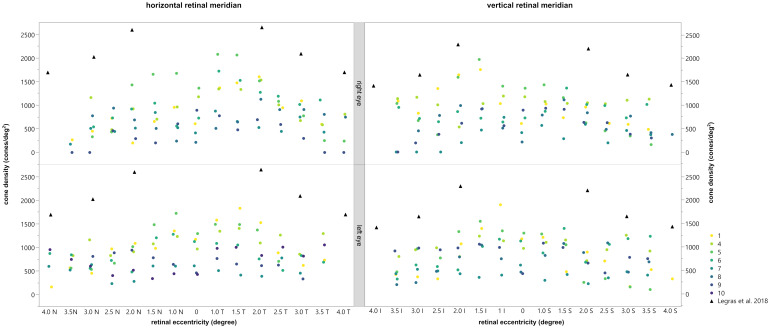
Comparison of the cone density with the normal values obtained by Legras et al., 2018 (27) (black triangles). The horizontal (left) and vertical (right) meridian of the right (top) and left (bottom) eye are shown for each subject, depicted by different colors. Permission of Legras et al. was officially obtained.

The density of the cone photoreceptors was analyzed at the fovea and at each 0.5 degree from 1–4 degree of eccentricity in both vertical and horizonal directions. The cone density per degree^2^ was calculated within these 80x80 pixels regions of interest. In **Figure 5**, examples are provided to illustrate the locations where cone density was measured in two patients. Seven regions of interest are shown with a higher magnification to provide a better insight into the photoreceptor mosaic along the horizontal and vertical meridian between 0 and 3 degrees. One patient with an advanced stage of retinitis pigmentosa and another patient with a milder progression are depicted. The results are shown in **Figure 6**, along with normative data from Legras et al., 2018 (27), who also analyzed the cone density and arrangement with AO in a healthy population of 109 subjects. Compared to healthy individuals and the normative data the cone density of the patients was severely reduced. Furthermore, a comparison with the outer nuclear layer (ONL) thickness observed in SD-OCT revealed its relation to the reduction in photoreceptor density in Usher patients. If the outer nuclear layer was thinned, and the inner segment/outer segment (IS/OS) line was interrupted, the cone mosaic was also disturbed, leading to a reduction in cell density.

In general, the calculated cone density was highest for patients 1, 4, 5, and 6 and lowest for patients 7, 8, 9 and 10. In patient 2 and 3 no cone density could be determined due to lower image quality. As already shown and described patients 1, 4, 5, and 6 had reduced mfERGs whereas patients 3, 7, 8, 9 and 10 had no remaining responses. Thus, there appeared to be a correlation between the cone density and the magnitude of the mfERG amplitude.

**Figure 7:**
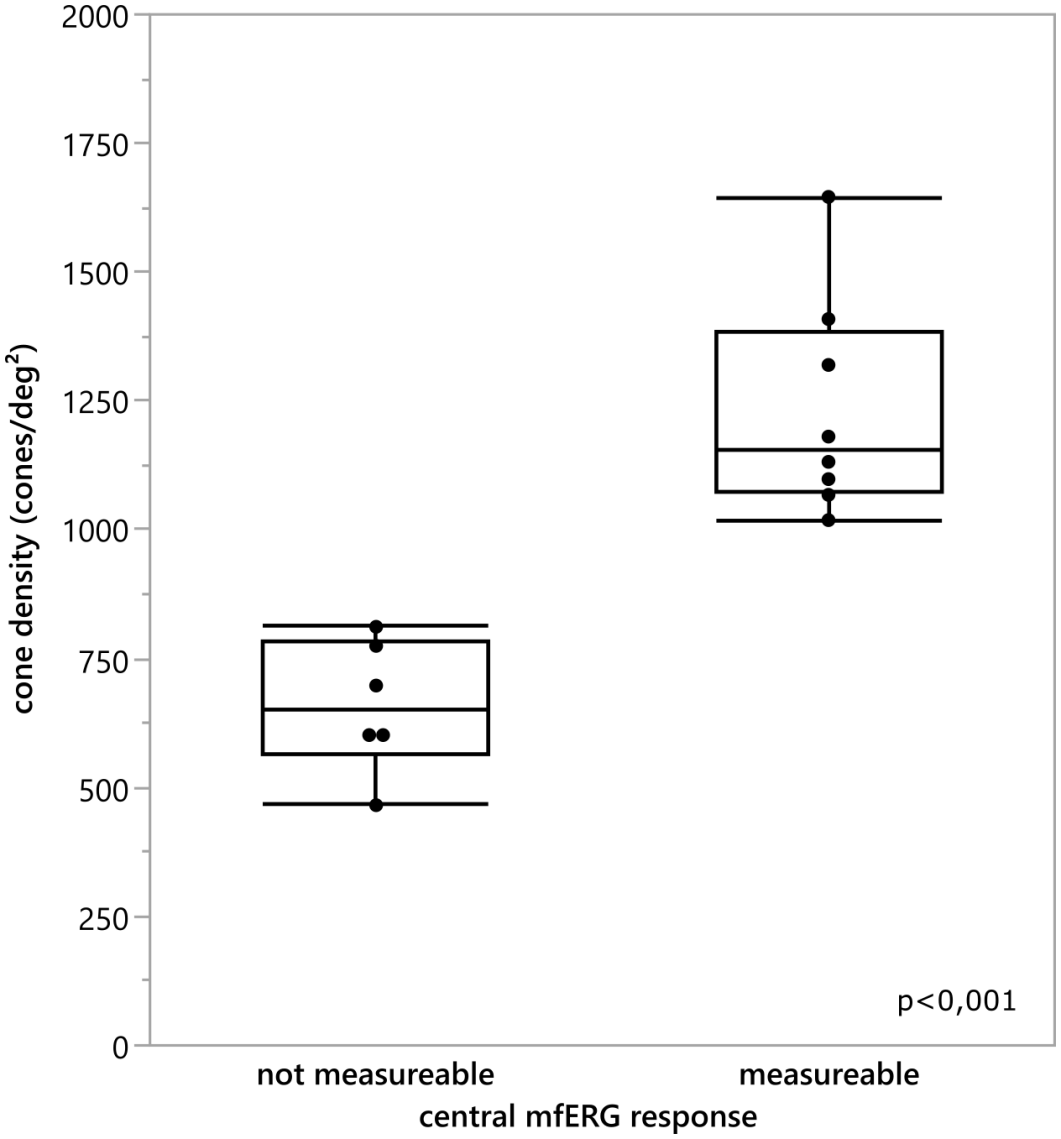
Comparison of the mean cone density inside the central 3°for every eye and the classification if the central 3 degree mfERG response was measurable. There was a statistically significant difference between the cone density of the eyes with an detectable mfERG response and the eyes without a measurable mfERG response t(11.628) = 6.295, p <0.001.

To analyze this in more detail we compared the cone density within the central 3 degrees with the central 3 degrees (radius 1.5 degree) response of the multifocal ERG. For the mean cone density the central nine ROI’s inside the eccentricity of 1.5 degree were calculated and averaged. The comparison revealed a statistically significant difference between the mean cone density of eyes with a measurable mfERG response and eyes without a measurable mfERG response [T (11.628)=6.295,p<0.001 (tested by the Welch test; see **Figure 7**)]. Patients 2 and 3 were excluded from the analysis due to the poor image quality of the AO images, which prevented us from making a prediction about the cone density.

**Figure 8:**
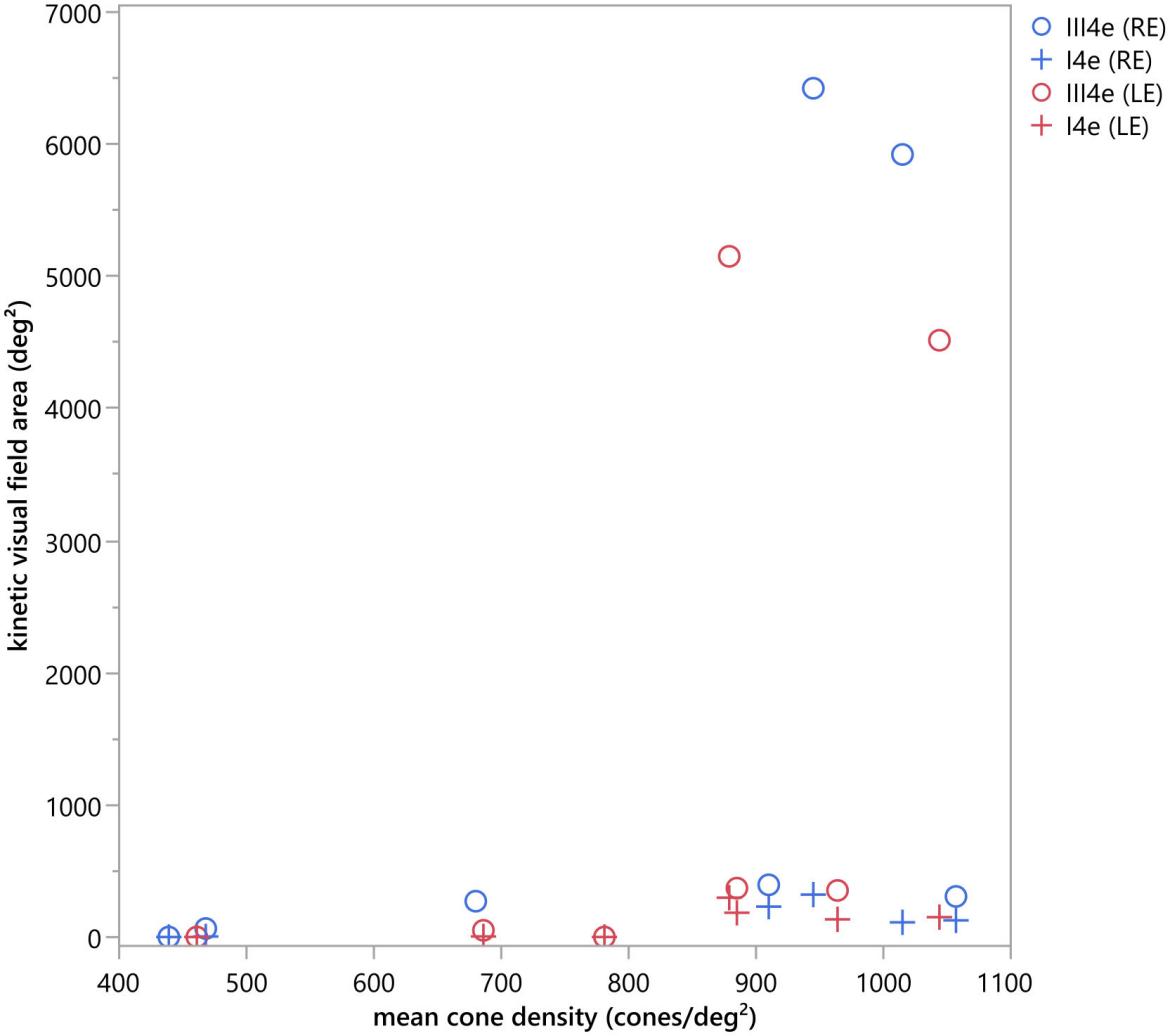
Scatter plot of the kinetic visual field area (deg^2^) measured with the III4e and I4e in comparison to the mean cone density (cones/deg^2^). No statistically significant correlation could be detected for the kinetic visual field area tested by the III4e (ρOD=0.75, pOD=0.052; ρOD= 0.685, pOS=0.090) and the I4e (ρOD=0.543, pOD=0.266; rOS=0.647, pOS=0.116).

The visual field area was compared with the mean cone density using the Pearson (r) correlation, or Spearman correlation (ρ) when the data was non-normally distributed, tested by Shapiro Wilk test (p<0.05). For the mean cone density, the cone density of all measurable ROIs was averaged. The correlation between the cone density and the visual field size was not statically significant (compare scatter plot in **Figure 8**), neither for the visual field area tested by the III4e (ρ_OD_=0.75, p_OD_=0.052; ρ_OD_= 0.685, p_OS_=0.090) nor for the area tested by the I4e (ρ_OD_=0.543, p_OD_=0.266; r_OS_=0.647, p_OS_=0.116).

## Discussion

Our results show that the cone density in patients with RP related to Usher Syndrome using the adaptive optics flood illumination retina camera was decreased compared to previously published data on healthy eyes (27). Photoreceptor cell death was seen as a low cone density, along with disorder of the cone mosaic (**Figures 4**, **5**). It is known that the cones within the central 2 degrees area cannot be resolved with AO due to the limit of resolution of the device. However, because a central loss of cones is expected in RP patients with Usher Syndrome, making the remaining cones detectable, the visualization and measurement of cell density could be established in the central fovea and at 1 degree. Therefore, we could observe visible cones in the fovea in the early stages of the disease, as well as a loss of cones outside the fovea, seen as blurry, dark patches. However, due to the anatomical distribution of the cones (28), we were expecting a much higher cone density in the central area, especially in the patients with a still good preserved visual acuity.

There are other reports that have examined the cone mosaic in RP using AO with similar patterns found as here (13, 29, 30). Gale et al. (29) categorized the changes in cone reflectivity into hypo-reflective blurred cone-like structures, higher frequency disorganized hyper-reflective spots and lower frequency hypo-reflective spots, which mirror progressive phases of cone degeneration observed using SD-OCT and FAF, as also shown by Tojo et al. (30) and Ueno et al. (31). This was also obvious in our study: The blurry patches and cone density matched the cone photoreceptor degeneration patterns shown by the SD-OCT and FAF imaging (**Figures 2**, **3**). Gale et al. (32) have also shown that the repeatability of the results in RP patients is the same as that of control subjects.

High-resolution measurements of the cone mosaic performed with the confocal AOSLO (17, 33–36), which measures aberrations from the entire scanned area of the retina including the fovea, confirm these findings. Even if the outer retinal architecture in the SD-OCT is normal, the cone density has been shown to range from normal to severely reduced in RP patients (17, 19, 33, 34). A study investigating RP patients with Usher syndrome has further shown that the foveal cone density determined with an AO-SLO can be decreased before visible changes in the SD-OCT or a decline in visual function (36). This highlights the sensitivity of the high-resolution AO imaging and lends weight to studies demonstrating that visual acuity and sensitivity measurements are relatively poor markers for the severity of retinal degeneration compared to high resolution imaging of foveal cone mosaic (11).

Nevertheless, one of our limitations is that when using the rtx1 compared to an AOSLO, the cone density may be underestimated due to poor image quality or reflectivity of the cones. It is not possible to distinguish between not waveguiding cells and dark spots. Furthermore, due to the lack of information on patients’ eye length, the cell density could only be expressed using cones per degree^2^ (visual units).

Moreover, in this study, retinal areas within the hyperautofluorescence in FAF image indicated normal outer retinal structures, whereas the AO-FIO images displayed increased spacing in the cone mosaic, as previously found with, AO-SLO imaging (26) and in descriptive analysis (15).

The ERGs were extinguished in 7 patients and reduced in 3 patients confirming the report by Stingl et al. (37) that the ERG is abnormal in Usher patients. Our results revealed a correlation between the mfERG results and the other imaging techniques. The mfERG has been shown to reliably provide even small remaining responses in the macular region of RP patients (38–40) and was also reduced in all our Usher patients. This study could confirm that the cone density of the central retina was in most cases significantly correlated (**Table 1**) to the mfERG response in the central 3° of the retina. When the cone density measurements from AO retinal images were compared with the mfERG recordings from corresponding areas, the analysis indicated that there was a clear correlation between the strength of mfERG responses and the cone density measurements, i.e., lower amplitudes were associated with lower cone density measurements confirming the results of Choi et al. (13) using AO images. Granse et al. (38) have demonstrated that mfERG and multifocal visual evoked potentials may be more useful for examining remaining visual function in RP patients than visual field measurements. These findings are consistent with the unspecific remaining extent of the visual fields measured in this study.

It has been shown that when corrected for age, the preserved kinetic visual field is significantly larger in USH2 than in USH1 (37) and patients with USH1 have vastly reduced visual fields already at an early age. A direct comparison of these two cohorts of different sizes and different clinical phenotypes is a limitation of this analysis. However, the heterogeneity of the patients inside our cohorts was high. For example, the 60-year-old USH1 patient 2 still had a comparatively large visual field, preserved scotopic ERG and his retinal layering was more widely preserved than the one of patient 6 (USH2). Due to this heterogeneity, we believe that these ten patients could be compared within the context of their phenotypic expression.

In conclusion, we find that FI-AO combined with SD-OCT and FAF imaging are useful and sensitive clinical imaging methods for examining photoreceptor alterations due to RP, although there are clear limitations of AO techniques in the clinical routine. An accurate quantification of structural alterations in the retina is fundamental for monitoring the success of future therapeutical interventions (41).

## Data availability statement

The raw data supporting the conclusions of this article will be made available by the authors, without undue reservation.

## Ethics statement

The studies involving humans were approved by Ethics Committee of the University of Tuebingen. The studies were conducted in accordance with the local legislation and institutional requirements. The participants provided their written informed consent to participate in this study. Written informed consent was obtained from the individual(s) for the publication of any potentially identifiable images or data included in this article.

## Author contributions

MK: Conceptualization, Formal analysis, Investigation, Methodology, Visualization, Writing – original draft, Writing – review & editing. SK: Writing – review & editing. KrS: Conceptualization, Formal analysis, Writing – review & editing. FN: Conceptualization, Investigation, Writing – review & editing. KaS: Funding acquisition, Writing – review & editing. FK: Formal analysis, Investigation, Writing – original draft.
